# Body Mass Index and Total Symptom Burden in Myeloproliferative Neoplasms Discovery of a U-shaped Association

**DOI:** 10.3390/cancers12082202

**Published:** 2020-08-06

**Authors:** Sarah Friis Christensen, Robyn Marie Scherber, Nana Brochmann, Martin Goros, Jonathan Gelfond, Christen Lykkegaard Andersen, Esben Meulengracht Flachs, Ruben Mesa

**Affiliations:** 1 Department of Hematology, Zealand University Hospital, 4000 Roskilde, Denmark; nana.brochmann@gmail.com; 2 Department of Hematology/Oncology, UT Health San Antonio MD Anderson Cancer Center, San Antonio, TX 78229, USA; MesaR@uthscsa.edu; 3 Hematologic Malignancies, Incyte Corporation, Wilmington, DE 19803, USA; 4 Department of Population Health Sciences, UT Health San Antonio, San Antonio, TX 78229, USA; goros@uthscsa.edu (M.G.); gelfondjal@uthscsa.edu (J.G.); 5 Department of Hematology, University Hospital of Copenhagen at Rigshospitalet, 2100 Copenhagen, Denmark; christen.andersen@sund.ku.dk; 6 Department of Occupational and Environmental Medicine, Bispebjerg University Hospital, 2400 Copenhagen, Denmark; esben.meulengracht.flachs@regionh.dk

**Keywords:** Philadelphia-negative myeloproliferative neoplasms (MPN), body mass index, symptom burden, quality of life, chronic inflammation

## Abstract

Elevated body mass index (BMI) is a global health problem, leading to enhanced mortality and the increased risk of several cancers including essential thrombocythemia (ET), a subtype of the Philadelphia-chromosome negative myeloproliferative neoplasms (MPN). Furthermore, evidence states that BMI is associated with the severity of symptom burden among cancer patients. MPN patients often suffer from severe symptom burden. The purpose of this study was to examine whether deviations from a normal BMI in an MPN population are associated with higher symptom burden and reduced quality of life (QoL). A combined analysis of two large cross-sectional surveys, the Danish Population-based Study, MPNhealthSurvey (*n* = 2044), and the international Fatigue Study (*n* = 1070), was performed. Symptoms and QoL were assessed using the validated Myeloproliferative Neoplasm Symptom Assessment Form (MPN-SAF). Analysis of covariance was used to estimate the effects of different BMI categories on symptom scores while adjusting for age, sex, and MPN subtype. A U-shaped association between BMI and Total Symptom Burden was observed in both datasets with significantly higher mean scores for underweight and obese patients relative to normal weight (mean difference: underweight 5.51 (25.8%), *p* = 0.006; obese 5.70 (26.6%) *p* < 0.001). This is an important finding, as BMI is a potentially modifiable factor in the care of MPN patients.

## 1. Introduction

Body mass index (BMI) is a key global health issue worldwide, as the prevalence of obesity has increased to a pandemic extent, and obesity has become one of the leading risk factors for premature death [1,2]. Furthermore, comprehensive research documents an association between obesity and the increased risk of multiple adverse health outcomes [3] including diabetes, cardiovascular diseases [2,3,4,5,6], and several common cancers (e.g., colon cancer, gastric cancer, breast cancer) [7,8,9,10]. Interestingly, recent studies indicate that obesity might increase the risk of essential thrombocythemia (ET), a subtype of the myeloproliferative neoplasms, as well [11,12,13,14,15]. Besides ET, Philadelphia-negative myeloproliferative neoplasms (MPN) encompass polycythemia vera (PV), myelofibrosis (MF), and MPN unclassified (MPN-U). They are acquired clonal hematologic cancers characterized by a state of chronic inflammation [16,17]. Through their lifelong course of disease, MPN patients are at risk of developing splenomegaly, vascular complications, bone marrow failure and progressing to acute myeloid leukemia [18], and in their daily lives, the patients often suffer from debilitating symptoms including fatigue, pruritus, abdominal discomfort, night sweats, and early satiety [19]. Inflammation may represent a key component in several of these symptoms [20]. Like MPN, obesity is known to trigger a state of chronic inflammation with elevated levels of pro-inflammatory cytokines [21,22,23,24,25,26,27], but how obesity affects symptom burden among MPN patients is largely unknown [28,29,30,31]. However, a broad body of literature evidences that an elevated BMI is associated with increased symptom burden and reduced health-related quality of life (HRQoL) [32,33,34,35,36,37,38,39,40]. Bearing in mind the rapidly increasing prevalence of obesity and the multiple adverse effects of MPN symptoms such as compromised social functioning, physical activity, self-reliance, productivity, and reduced quality of life, it is paramount to gain further knowledge in an MPN context [19,41]. Accordingly, the aim of this study is to examine the association between BMI and symptom burden and quality of life, respectively.

## 2. Results

### 2.1. Patient Demographics

In total, 3114 patients (1852 females, 1256 males) were included in the combined analysis, with 2044 patients from the Danish MPNhealthSurvey and 1070 patients from the International Fatigue Study (Table 1). Patients were of typical age (mean 65.6 years) for the disorder with a distribution of participants consisting of 1047 patients with ET (33.7%), 1301 patients with PV (41.8%), 330 patients with MF (10.6%), and 433 with MPN-U (13.9%). The majority of patients had a duration of disease of a least a few years (>3y [*n* = 2242, 71%], 1–3 y [*n* = 623, 20%], ½–1 y [*n* = 170, 5.8%], <½ y [*n* = 67, 2.2%]). Comparing patients from the Danish MPNhealthSurvey and the Fatigue Study, Danish patients were significantly older (69.2 y vs. 60.9 y, *p* < 0.0001), a smaller proportion was female (56.5%, vs. 67.3%, *p* < 0.0001) and fewer patients were diagnosed with MF (3.3% vs. 24.6%, *p* < 0.0001). Time since MPN diagnosis was similar between the studies except for a greater proportion of newly diagnosed patients in the Fatigue Study (5.1% vs. 0.6%, *p* < 0.0001%). In both the US and the Danish dataset, patients with *Normal Weight* were most frequent (DK/US = 52.4/46.3%), followed by *Overweight* patients (DK/US = 31.7/30.5%), *Obese* patients (DK/US = 13.2/21.7%), and least frequent *Underweight* patients (DK/US = 2.7%/1.5%). The mean BMI in the study population was 25.7, with no significant differences in mean BMI when stratifying by MPN subtype (Table S1). In contrast, we found an overall difference between the MPN subtypes with MF patients reporting a significantly higher TSS compared with ET patients (25.2 vs. 21.9, *p* < 0.05), especially underweight MF patients reported a high symptom burden (mean TSS = 41.0) (Table S2)

### 2.2. Body Mass Index and Symptom Burden

**Figure 1:**
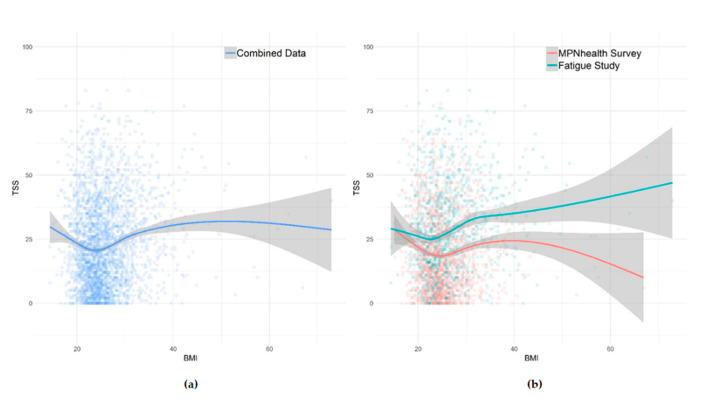
Association between Body Mass Index (BMI) and Total Symptom Score (TSS). TSS was calculated for all respondents completing at least six of 10 TSS survey items. A high score represents high total symptom burden: (**a**) combined data; (**b**) by study.

**Figure 2:**
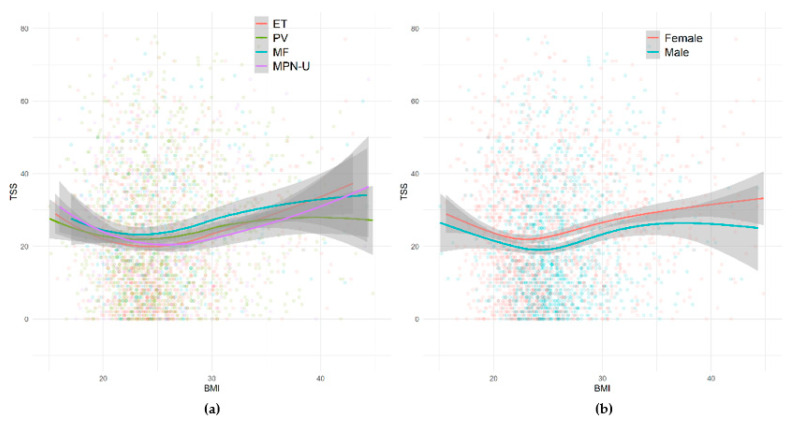
Association between BMI and TSS by MPN subtype (**a**) and gender (**b**), respectively. TSS was calculated for all respondents completing at least six of 10 TSS survey items. A high score represents high total symptom burden.

A U-shaped association between BMI and Total Symptom Score (TSS) was demonstrated, with a higher symptom burden among MPN patients who are underweight or obese compared with patients in the normal weight BMI category; a finding that was consistent in both the merged analysis and when looking at the two study populations separately (Figure 1). Likewise, the pattern remained when stratifying by gender and MPN subtype, respectively (Figure 2). 

Furthermore, the U-shaped pattern (underweight > normal weight < obese) persisted for nearly all MPN-SAF items when evaluating the mean symptoms score for each symptom individually; fatigue (4.36, 4.08, 5.25; *p* < 0.001), early satiety (3.86, 2.58, 2.91; *p* < 0.001), abdominal pain (1.75, 1.27, 1.80; *p* < 0.001), abdominal discomfort (2.26, 1.78, 2.33; *p* < 0.001), inactivity (3.12, 2.38, 3.66; *p* < 0.001), headache (1.90, 1.76, 2.38; *p* < 0.001), concentration problems (3.09, 2.69, 3.59; *p* < 0.001), dizziness (2.71, 2.24, 2.82; *p* < 0.001), numbness (2.83, 2.25, 3.19; *p* < 0.001), insomnia (3.54, 3.04, 3.97; *p* < 0.001), sad mood (2.67, 2.40, 3.17; *p* < 0.001), cough (2.56, 1.61, 2.37; *p* < 0.001), bone pain (2.40, 1.84, 2.96; *p* < 0.001), fever (0.42, 0.31, 0.42; *p* = 0.057), and quality of life (3.61, 2.90, 3.50; *p* < 0.001) (Table 2). 

In line with these findings, symptom prevalence (MPN-SAF score ≥ 1) by BMI also showed the characteristic U-shape for the vast majority of symptoms (underweight > normal weight < obese); early satiety (76.8%, 62.4%, 67.7%; *p* = 0.018), abdominal pain (44.9%, 38.2%, 50.1%; *p* < 0.001), abdominal discomfort (59.4%, 50.0%, 60.5%; *p* < 0.001), inactivity (69.2%, 61.5%, 76.7%; *p* < 0.001), concentration problems (68.6%, 63.5%, 72.6%; *p* = 0.003), dizziness (70.0%, 60.7%, 66.5%; *p* = 0.057), numbness (59.4%, 54.5%, 67.2%; *p* < 0.001), insomnia (71.0%, 68.2%, 76.8%; *p* = 0.004), sad mood (71.4%, 62.0%, 72.7%; *p* < 0.001), cough (64.3%, 47.0%, 58.2%; *p* < 0.001), itching (54.3%, 53.4%, 66.6%; *p* < 0.001), bone pain (50.0%, 45.9%, 60.9%; *p* < 0.001), and fever (20.3%, 12.4%, 15.8%; *p* = 0.047) (Table 3).

The U-shaped pattern did not exist for weight loss; obese patients had both the lowest average score and prevalence of this symptom while underweight patients had the highest score. Increasing means with increasing BMI categories were observed for night sweats (2.33, 2.40, 2.72, 3.28 *p* < 0.001) and sexuality problems (3.29, 3.36, 3.91, 4.37, *p* < 0.001) with obese patients reporting the greatest severity for these symptoms. Likewise, the same pattern was seen for symptom prevalence (night sweats: 52.9%, 59.4%, 61.5%, 69.5% *p* < 0.001; sexuality problems: 55.4%, 62.7%, 67.7%, 71.0%, *p* < 0.001) (Table 2 and Table 3). 

### 2.3. Adjusted Differences in MPN-SAF Symptom Score

As shown in Table 4, adjusting for age, gender, and MPN subtype did not affect the observed association between BMI and symptoms markedly. For each of the single symptoms except weight loss, obese patients reported an average mean between 12.8–53.3% higher relative to patients with normal BMI. A notable difference in the mean of more than 25% existed for bone pain (53.3%, 0.98, *p* < 0.001), inactivity (51.3%, 1.22 *p* < 0.001), cough (47.8%, 0.77, *p* < 0.001), itching (41.1%, 0.86, *p* < 0.001), numbness (40.0%, 0.90, *p* < 0.001), abdominal pain (36.2%, 0.46, *p* < 0.001), night sweats (33.8%, 0.81, *p* < 0.001), sad mood (26.7%, 0.64, *p* < 0.001), total symptom score (26.8%, 5.73, *p* < 0.001), concentrations problems (26.4%, 0.71, *p* < 0.001), abdominal discomfort (25.8%, 0.46, *p* < 0.001), sexuality problems (25.7%, 0.89, *p* < 0.001), and insomnia (25.3%, 0.77, *p* > 0.001). Comparing obese and normal weight patients, fever was the only symptom that did not differ significantly. As mentioned, the patients in the underweight BMI category reported a worse symptoms score compared with patients with normal weight. Underweight patients had a more than 25 percent increased symptom score for cough (57.8%, 0.93, *p* = 0.002), early satiety (48.4%, 1.25, *p* < 0.001), inactivity (31.9%, 0.76, *p* = 0.026), total symptom score (25.1%, 5.37, *p* = 0.007), and, as anticipated, a much higher severity of weight loss (176.6%, 2.19, *p* < 0.001). Finally, in regard to overweight patients, the same pattern was observed, however, the differences in means compared with patients with normal weight were smaller with fewer significant findings.

## 3. Discussion

For many, being diagnosed with MPN means facing a life with substantial symptom burden throughout the lifelong disease course [19,41,42,43,44]. As MPN symptoms impact patient QoL, treatment and survival, alleviating symptom burden is fundamental in MPN therapy. During the last decade, our understanding of the MPN symptom profile (i.e., symptom prevalence and symptom severity) has increased markedly, due to the development of specific MPN symptom scoring tools [42,43]. However, our understanding of the multifactorial causes of the different disease-related symptoms are currently sparse, and further research uncovering contributing factors are crucial. Accordingly, this is the first study to investigate if total symptom burden (TSS), individual MPN symptoms, and quality of life are associated with BMI. 

In this collaborative international study using the combined data from two comprehensive cross-sectional health-related quality of life studies, we found several interesting results. Most importantly, we discovered a U-shaped distribution of total symptom burden with the nadir centered around normal body weight. That is, when compared with MPN patients in the normal BMI category, underweight and obese patients had a significantly higher total symptom score (TSS). This novel finding was consistent both in the merged data analysis and by study as well as stratified by gender and MPN subtype, respectively. Furthermore, the U-shaped pattern was also found for several of the single symptoms and quality of life. 

Supporting our findings are two recently published abstracts. A cross-sectional study focusing on nutrition among MPN patients showed a similar mean BMI (=25.9) that did also not differ significantly among MPN subtypes. Furthermore, the study found a BMI > 25 to be significantly associated with higher TTS compared with a BMI < 25 [29]. In the nutrition study, when stratifying by MPN subtype, higher symptom burden among overweight/obese patients persisted, though not reaching statistical significance in patients with ET. Likewise, a study of the impact of weight on symptom burden in MPN patients participating in an online yoga intervention indicated higher symptom burden (TSS) at the baseline among patients with BMI > 25 (*p* = 0.06) [31]. On the contrary, in a retrospective study of 380 myelofibrosis patients, BMI did not influence the achievement of symptom response during treatment with Ruxolitinib [30]. 

The association between BMI and the most important MPN-related symptoms have never been examined before, individually. We found that obese patient (BMI > 30) compared with patients in the normal weight category (BMI 18.5–25) had significantly higher mean scores for the entire spectrum of symptoms with fever and weight loss as the only exceptions. These findings were present both in the raw data and in the analysis adjusted for age, sex, and MPN subtype. Similarly, overweight MPN patients (BMI 25–30) had higher symptom mean scores for nearly all symptoms, as compared with normal weight MPN patients. However, the observed differences were of smaller magnitude, and fewer differences reached statistical significance. Our results align with previously published data in other cancer populations and the general population, demonstrating that obese patients suffer from increased symptom burden. For example, studies in patients with breast cancer report higher BMI to be associated with increased pain [45,46], sadness [33], numbness [33], fatigue [33,47], and other treatment-related symptoms [48]. Moreover, a comprehensive review of the effect of obesity on health outcomes suggests robust evidence of obesity being associated with reduced sexual functioning which is in accordance with our findings [49]. Likewise, epidemiologic research has shown obese individuals more often suffer from headache, as compared with those of normal weight [50].

One of the most striking findings in our study was the U-shaped pattern of body mass index and overall quality of life with normal weight MPN patients reporting the best quality of life. To our knowledge, comparable studies in MPN patients have not been published. However, consistent with our results, health-related quality of life studies in healthy people, cancer patients, and cancer survivors suggest an inferior quality of life among obese persons [32,35,40,51,52,53,54,55] of all ages [37,56,57,58,59]. Noteworthy, a health survey from England of >14.000 persons showed a similar U-shaped pattern of HRQoL with the best HRQoL close to a BMI of 25 with worse HRQOL for both higher and lower BMI values [36]. 

The pathogenesis behind the increased symptom burden in obese and underweight MPN patients, respectively, is probably distinct. However, inflammation is likely to play a key role in both pathways. Chronic low-grade inflammation is a hallmark feature of the MPN disease as the JAK2V617F mutation induces constitutive activation of the Janus kinase cascade, resulting in increased levels of pro-inflammatory cytokines [20,60,61,62,63], and recent studies indicate that specific MPN symptoms are associated with specific markers of inflammation [20,61,62,63]. Equally, it is well established that obesity leads to systemic chronic inflammation [21,22,23,24,25,26,27]. The elevated inflammatory status in obese persons originates from infiltrating immune cells, in particular, macrophages in the adipose tissue with a altered production of pro-inflammatory molecules, “adipokines” [64]. We speculate, that a contributing factor to higher symptom burden, observed in our study among obese patients as compared to normal weight patients, is the accumulate inflammatory effect of being obese and having an MPN disease. Our hypothesis is supported by research in humans and animals showing that several of the increased pro-inflammatory molecules in the two conditions are overlapping (e.g., TNF-α, IL1, IL-6, MCP-1, CRP) [24,26,27,60,61,64,65]. 

Consistent with previous research highlighting fatigue as one of the most prominent symptoms in MPN [19,42], we found MPN patients across all BMI categories often experience fatigue. However, in the obese category, the prevalence was even higher, with almost 90% of obese MPN patients reporting fatigue. The pro-inflammatory marker, TNFα, has been shown to induce fatigue in other malignancies [66] and interestingly, TNFα is elevated in both MPN patients [61] and obese persons in the general population [27,67]. Thus, a potential additive effect of TNFα might be a contributing cause of the pronounced fatigue among obese MPN patients in the current study, however this hypothesis remains to be explored. Obesity-related inflammation is thought to be a key feature of insulin resistance and development of type 2 diabetes [27,68], a comorbidity also known to be associated with fatigue [69,70] and therefore another possible contributing cause of the high prevalence of fatigue. In addition, research in cancer patients suggests fatigue is associated with higher levels of depressive symptoms [71,72] and in agreement with this, MPN patients in the obese BMI category also reported higher prevalence and severity of “sad mood”. The exact pathophysiological causes are yet to be fully determined, however, circulating levels of cytokines including IL6 have been linked to fatigue and depressed mood [73,74,75], and as IL6 is elevated in both obesity and MPN further illumination of the underlying mechanisms is crucial. Apart from being associated with depression, levels of IL6 along with IL1 and CRP have been shown to be increased in patients with cognitive impairment [76,77,78,79] and in obese persons [22,24,26], respectively. With those findings in mind, it is of great interest that obese MPN patients had a 26% higher mean score for concentration problems compared with normal weight MPN patients. Abdominal-related symptoms including abdominal discomfort, pain, and early satiety are commonly reported among MPN patients, but also those complaints were more frequent and severe among obese MPN patients. Splenomegaly is an important cause of abdominal-related symptoms, however, it is more difficult to diagnose by palpation in people with abdominal obesity. Furthermore, the enhanced inflammation in obese MPN patients may also be of importance in the increased severity of abdominal-related symptoms, as cytokines can induce the enlargement of the spleen and may worsen abdominal pain, and pain in the general, by cytokine-induced nerve hyperstimulation [20,61]. 

Interestingly, not only obese MPN patients but also underweight patients reported increased total symptom burden with higher severity of the majority of single symptoms. Whilst many have studied the impact of underweight on mortality, little is known about underweight MPN patients’ health-related quality of life, however, studies in the general population including patients with chronic diseases likewise demonstrate a deterioration of health-related quality of life among underweight individuals [39,80,81]. 

Underweight is described as a rather heterogeneous condition, which can be found in otherwise healthy persons, patients suffering from malnutrition or eating disorders as well as chronic diseases and cancer [80,82]. In an MPN context, undesired weight loss is a well-known complication, particularly in regards to MF [19]. However, the mean BMI of the MF patients was not significantly lower than the other MPN subtypes, which might be due to selection bias, i.e., underweight patients may be too sick to overcome participating in a questionnaire study. Furthermore, only few underweight MF patients participated in the present study. Of notable interest, underweight MF patients reported markedly higher symptom burden compared with all other patients across MPN subtypes and BMI categories, indicating that their low BMI might be due to cachexia. A syndrome characterized by the loss of skeletal muscle and fat mass [83,84].

Along with other constitutional symptoms, cachexia may be a sign of disease progression [30,85]. Accordingly, we found, that constitutional symptoms such as weight loss and fever were most prevalent among underweight patients. Cachexia is a multifactorial syndrome, with several triggering causes including low nutritional intake due to bothersome splenomegaly [84], which might be a plausible explanation of the high level of “early satiety” reported by the underweight MPN patients in the present study. Besides splenomegaly, it is currently assumed that both the metabolic disturbances and the constitutional symptoms in MF, at least partly, are a result of a cytokine-driven systemic inflammatory state induced by the dysregulation of the JAK-STAT pathway [84,86]. The JAK-STAT pathway is vital in the regulation of pro-inflammatory cytokines such as IL-6 and TNF-α of which both have been shown to be involved in the modulation of cachexia [84,87]. Furthermore, the inflammatory marker, C-reactive protein, is increased in patients suffering from cancer-cachexia [84]. 

Bearing in mind that individuals with very high or low body weights often have elevated inflammatory levels and that the present study demonstrate reduced health-related quality of life in underweight and obese patients, and finally that inflammation is suggested to contribute to disease progression by promotion of neoplastic stem cell growth and suppression of normal hematopoiesis, interventions trying to reduce inflammation are vital. Several studies indicate that an anti-inflammatory diet and exercise have the potential to attenuate inflammation [88,89,90,91,92,93] and improve HRQoL [40,47,53,55,94,95,96,97]. Within the MPN field, only recently studies have started to investigate these promising non-pharmacological interventions to dampen inflammation and alleviate symptom burden [98]. A pilot study of yoga as supplementary care in MPN found that yoga was beneficial, leading to a significant reduction in total symptom burden and, furthermore, 75% of the participants felt yoga was helpful in coping with their MPN symptoms [99]. On the contrary, a five-day interdisciplinary exercise-based rehabilitation intervention followed by a 12-week self-exercising program, including 45 MPN patients, did not find a significant impact on symptom burden, however, the median adherence to self-exercise was low, which might explain the lack of impact [100]. Interestingly, studies in other hematological cancers suggest a positive impact of physical activity on a variety of patient-reported outcomes [101]. Hence, more research is needed to make evidence-based recommendations for exercise as a symptom management strategy in MPN treatment. Additionally, only preliminary data exist in regard to the potential of anti-inflammatory diet as a tool in MPN care; a cross-sectional study of nutrition among 1329 MPN patients revealed that pro-inflammatory food as fast food and refined sugar was associated with an increased total symptom score. The survey further revealed that 96.2% of MPN patients were willing to restrict their diet if it helped reduce their symptoms [98,102]. 

Some limitations of the current study should be noted. Firstly, the self-reported outcomes and measures lack the validation of MPN diagnosis and BMI. Secondly, the cross-sectional design makes the study unable to establish causality between BMI and symptom burden; although BMI is likely to influence HRQL, the direction of causality may also be the opposite, e.g., quality of life or fatigue may impact eating behavior and physical activity. Furthermore, the Danish version of MPN-SAF has not been psychometrically validated, however, the questionnaire has been translated and linguistically validated by a professional translator according to international guidelines on the translation and cultural adaptation of patient-reported outcomes [103], and the MPN-SAF is an internationally recognized valid questionnaire designed specifically to MPN patients [42]. Finally, the collaborative study on BMI was not a part of the originally planned research questions, hence, the current study must be characterized as explorative, and the results must be confirmed in a future study. Despite these limitations, we consider the findings in this study to be of high clinical importance, as the study, given the large number of participating MPN patients, provides high significant results. Moreover, the results are very consistent across different types of symptoms, which support the hypnotized association between BMI and symptom burden. Another strength of the current study is the good coverage of all BMI categories, proving results that cover the majority of MPN patients.

## 4. Materials and Methods

### 4.1. Descriptions of Surveys

A comprehensive analysis of data from two large cross-sectional surveys of health-related quality of life in MPN patients was done by combining data from the Danish Population-based Study, MPNhealthSurvey (*n* = 2044) [104], with data from the international Fatigue Study (*n* = 1040) [28]. Both surveys are described in detail elsewhere, but in brief, the MPNhealthSurvey is a Danish nationwide population-based survey, where patients with an MPN diagnosis registered in the Danish National Patient Register (NPR) between 1977 (at the time of NPR initiation) and March 31, 2013, and who were alive on September 4, 2013, when the survey population was formed, were invited to participate [105]. The invitations were sent on September 11, 2013, and the survey period ended December 31, 2013. Patients were asked to complete a survey booklet received by mail or complete the same survey online. Information on addresses, age, and sex was retrieved from the Danish Civil Registration System [106]. The Fatigue Study is a comprehensive internet-based survey developed by MPN investigators and patients at the Mayo Clinic Survey Research Center. The survey was available online via multiple MPN-related web sites including MPN Voice, MPN Research Foundation, the MPN Forum, and their respective Facebook pages from late February to March of 2014. Participants were self-identified MPN patients who understood written English with the majority of participants residing in the US (65%).

The MPNhealthSurvey was approved by the The Danish Data Protection Agency (SJ-RO-02). In Denmark, approval from the National Committee on Health Research Ethics is not required for questionnaire studies (Committee Act section 14, 2) [107]. The completion of the survey was deemed as an agreement of consent from the respondents. The Fatigue Study was approved by The Mayo Clinic Institutional Review Board and the Ethics committee, as an online minimal risk study, patients completed a modified consent before accessing the survey. Both studies were conducted in accordance with the Declaration of Helsinki. 

### 4.2. Symptoms, Lifestyle Factors, and BMI Categories

In both surveys, symptom burden and quality of life were assessed utilizing the validated Myeloproliferative Neoplasm Symptom Assessment Form (MPN-SAF) in conjunction with the Brief Fatigue Inventory (BFI) [42]. Patients reported 18 MPN-related symptoms (worst fatigue (BFI score), early satiety, abdominal pain, abdominal discomfort, inactivity, concentration problems, dizziness, numbness, insomnia, sad mood, sexuality problems, cough, night sweats, itching, bone pain, fever, weight loss, and quality of life) on a scale of 0 (absent) to 10 (worst imaginable). For participants completing at least 6 of the 10 most representative MPN-SAF symptoms, a Total Symptom Score (TSS) was calculated by multiplying the average score across items by 10 to achieve a scaled score of 0 to 100 [43]. In addition, both surveys included questions regarding educational level and lifestyle factors such as tobacco use, alcohol intake, and physical activity. Furthermore, participants reported their current height and weight. BMI was calculated, and patients were split into underweight (<18.5), normal weight (18.5–24.9), overweight (25.0–29.9) and obese (≥30) in consensus with the prevailing WHO guidelines [108]. 

### 4.3. Statistical Analysis

We evaluated the associations of BMI both by study and combined. Kruskal–Wallis tests for continuous variables and chi-squared test and Fisher’s exact test for categorical variables. Analysis of covariance was used to estimate the effects of different BMI categories on symptom scores while adjusting for age, sex, and MPN subtype. We used locally weighted scatterplot smoothing (LOESS) to visualize the U-shaped BMI associations. All statistical testing was two-sided with a significance level of 0.05. The R (R Foundation for statistical computing, Vienna, Austria) statistical software was used within an accountable data analysis process [109].

## 5. Conclusions

We found a U-shaped relationship between BMI and total symptom burden, several single symptoms and not least QoL; a pattern that was consistent across countries and remained significant after adjusting for age, sex, and MPN subtype. Despite medicinal treatment, symptom burden remains a challenge, and due to the chronic nature of MPNs, patients may face several years of disabling symptoms and impairment in quality of life. Furthermore, underweight and obesity is often associated with elevated inflammatory levels and inferior survival. Recently, non-pharmacologic interventions targeting chronic inflammation have shown promising results. Bearing in mind that confounders may be present, the association between BMI and symptom burden is a critical finding, as BMI is a modifiable factor in the care of MPN patients with the potential of safe interventions to contribute to reduced symptom burden and improved QoL among MPN patients. Our findings need to be confirmed in future studies, and the impact of non-pharmacologic treatment modalities such as nutrition, weight alteration, and exercise as an adjunctive therapeutic strategy to diminish symptom burden in MPN need to be investigated in future trials.

## Figures and Tables

**Table 1:** MPN patients’ demographics by study.

Demographics	MPN Health Survey *n* = 2044	The Fatigue Study *n* = 1070	Total *n* = 3114	*p*-Value
Age (mean, sd)	69.0 (12.3)	58.8 (11.6)	65.5 (13.0)	<0.0001
Gender (N, %)				<0.0001
Female	1150 (56.3)	702 (66.0)	1852 (59.6)	
Male	894 (43.7)	362 (34.0)	1256 (40.4)	
MPN subtype (*n*, %)				<0.0001
ET	697 (34.1)	350 (32.8)	1047 (33.7)	
PV	878 (43.0)	423 (39.6)	1301 (41.8)	
MF	68 (3.3)	262 (24.6)	330 (10.6)	
MPN-U	401 (19.6)	32 (3.0)	433 (13.9)	
Disease duration (*n*, %)				<0.0001
<½ years	13 (0.6)	54 (5.1)	67 (2.2)	
½–1 years	136 (6.7)	43 (4.0)	179 (5.8)	
1–3 years	407 (19.9)	216 (20.2)	623 (20.0)	
>3 years	1488 (72.8)	754 (70.7)	2242 (72.1)	
BMI (BMI, sd)				<0.0001
<18.5	54 (2.6)	18 (1.7)	72 (2.3)	
18.5–24.9	1072 (52.4)	495 (46.3)	1567 (50.3)	
25.0–29.9	648 (31.7)	335 (31.3)	983 (31.6)	
≥30	270 (13.2)	222 (20.7)	492 (15.8)	

*MPN:* Philadelphia-chromosome negative myeloproliferative neoplasms.

**Table 2:** Symptom severity by BMI (mean, sd).

MPN-SAF Item	Underweight *n* = 72	Normal Weight *n* = 1567	Overweight *n* = 983	Obese *n* = 492	*p*-Value
Fatigue (BFI score)	4.36 (3.39)	4.08 (3.14)	4.26 (3.14)	5.25 (3.11)	<0.001
Early satiety	3.86 (3.05)	2.58 (2.70)	2.56 (2.62)	2.91 (2.70)	<0.001
Abdominal pain	1.75 (2.63)	1.27 (2.17)	1.34 (2.17)	1.80 (2.40)	<0.001
Abdominal discomfort	2.26 (2.66)	1.78 (2.45)	1.93 (2.44)	2.33 (2.64)	<0.001
Inactivity	3.12 (3.00)	2.38 (2.63)	2.65 (2.72)	3.66 (2.89)	<0.001
Headache	1.90 (2.87)	1.76 (2.57)	1.88 (2.58)	2.38 (2.90)	<0.001
Concentration problems	3.09 (3.12)	2.69 (2.82)	2.92 (2.88)	3.59 (3.12)	<0.001
Dizziness	2.71 (2.68)	2.24 (2.59)	2.32 (2.57)	2.82 (2.88)	0.001
Numbness	2.83 (3.24)	2.25 (2.82)	2.34 (2.85)	3.19 (3.15)	<0.001
Insomnia	3.54 (3.25)	3.04 (2.99)	3.19 (3.11)	3.97 (3.22)	<0.001
Sad mood	2.67 (2.74)	2.40 (2.71)	2.49 (2.71)	3.17 (2.88)	<0.001
Sexuality problems	3.29 (3.85)	3.46 (3.61)	3.91 (3.66)	4.37 (3.79)	<0.001
Cough	2.56 (2.98)	1.61 (2.36)	1.80 (2.46)	2.37 (2.80)	<0.001
Night sweats	2.33 (3.00)	2.40 (2.83)	2.72 (3.02)	3.28 (3.16)	<0.001
Itching	1.91 (2.48)	2.09 (2.74)	2.52 (2.96)	3.03 (3.12)	<0.001
Bone pain	2.40 (3.17)	1.84 (2.70)	2.08 (2.86)	2.96 (3.18)	<0.001
Fever	0.42 (1.12)	0.31 (1.13)	0.32 (1.03)	0.42 (1.30)	0.057
Weight loss	3.41 (3.52)	1.24 (2.34)	0.61 (1.66)	0.54 (1.71)	<0.001
Quality of life	3.61 (2.71)	2.90 (2.44)	3.08 (2.51)	3.50 (2.49)	<0.001
Total Symptom Score	26.65 (17.27)	21.39 (16.38)	22.57 (16.66)	27.95 (17.06)	<0.001

BMI: Body Mass Index, sd: standard deviation.

**Table 3:** Symptom prevalence ^1^ by BMI (*n*, %).

MPN-SAF Item	Underweight (*n* = 72)	Normal Weight (*n* = 1567)	Overweight (*n* = 983)	Obese (*n* = 492)	*p*-Value
Fatigue (BFI score)	54 (78.3)	1238 (80.3)	795 (82.1)	434 (89.5)	<0.001
Early satiety	53 (76.8)	961 (62.4)	610 (62.7)	331 (67.7)	0.018
Abdominal pain	31 (44.9)	590 (38.2)	397 (41.0)	244 (50.1)	<0.001
Abdominal discomfort	41 (59.4)	773 (50.0)	521 (53.8)	295 (60.5)	<0.001
Inactivity	45 (69.2)	932 (61.5)	609 (64.4)	366 (76.7)	<0.001
Headache	31 (44.9)	741 (47.7)	492 (50.4)	280 (57.4)	0.002
Concentration problems	48 (68.6)	979 (63.5)	645 (67.0)	352 (72.6)	0.003
Dizziness	49 (70.0)	937 (60.7)	618 (63.5)	326 (66.5)	0.057
Numbness	41 (59.4)	843 (54.5)	542 (55.8)	328 (67.2)	<0.001
Insomnia	49 (71.0)	1054 (68.2)	671 (68.9)	374 (76.8)	0.004
Sad mood	50 (71.4)	957 (62.0)	612 (62.9)	356 (72.7)	<0.001
Sexuality problems	36 (55.4)	943 (62.7)	642 (67.7)	340 (71.0)	0.001
Cough	45 (64.3)	726 (47.0)	488 (50.2)	285 (58.2)	<0.001
Night sweats	37 (52.9)	918 (59.4)	598 (61.5)	342 (69.5)	<0.001
Itching	38 (54.3)	822 (53.4)	581 (59.8)	327 (66.6)	<0.001
Bone pain	35 (50.0)	706 (45.9)	461 (47.6)	296 (60.9)	<0.001
Fever	14 (20.3)	191 (12.4)	146 (15.1)	77 (15.8)	0.047
Weight loss	44 (62.0)	493 (31.9)	174 (17.8)	78 (16.0)	<0.001
Quality of life	58 (82.9)	1224 (79.2)	783 (80.6)	411 (84.0)	0.123

^1^ Prevalence was defined as a symptom score greater than or equal to 1.

**Table 4:** Differences in symptom severity by BMI adjusted for possible confounders (age, gender, and MPN subtype).

MPN-SAF Item	Underweight ^1^			Overweight ^1^			Obese ^1^		
	Difference (%)	Mean Difference (CI95%)	*p*-Value (Mean)	Difference (%)	Mean Difference (CI95%)	*p*-Value (Mean)	Difference (%)	Mean Difference (CI95%)	*p*-Value (Mean)
Fatigue (BFI score)	10.0	0.41 (−0.32, 1.13)	0.269	4.9	0.20 (−0.04, 0.45)	0.098	23.3	0.95 (0.65, 1.26)	<0.001
Early satiety	48.4	1.25 (0.61, 1.9)	<0.001	0.4	0.01 (−0.20, 0.23)	0.893	12.8	0.33 (0.05, 0.6)	0.02
Abdominal pain	37.8	0.48 (−0.05, 1.01)	0.078	7.9	0.10 (−0.07, 0.28)	0.251	36.2	0.46 (0.23, 0.69)	<0.001
Abdominal discomfort	27.0	0.48 (−0.11, 1.07)	0.11	10.7	0.19 (−0.01, 0.39)	0.062	25.8	0.46 (0.21, 0.71)	<0.001
Inactivity	31.9	0.76 (0.09, 1.43)	0.026	10.5	0.25 (0.03, 0.48)	0.025	51.3	1.22 (0.94, 1.5)	<0.001
Headache	9.1	0.16 (−0.44, 0.77)	0.599	8.0	0.14 (−0.06, 0.35)	0.164	22.7	0.4 (0.15, 0.66)	0.002
Concentration problems	17.1	0.46 (−0.21, 1.13)	0.18	8.2	0.22 (0.00, 0.45)	0.054	26.4	0.71 (0.42, 1)	<0.001
Dizziness	20.5	0.46 (−0.17, 1.09)	0.149	5.8	0.13 (−0.08, 0.35)	0.214	23.2	0.52 (0.25, 0.79)	<0.001
Numbness	24.4	0.55 (−0.14, 1.25)	0.12	5.8	0.13 (−0.10, 0.37)	0.267	40.0	0.9 (0.6, 1.2)	<0.001
Insomnia	14.1	0.43 (−0.3, 1.16)	0.246	8.2	0.25 (0.01, 0.50)	0.042	25.3	0.77 (0.46, 1.08)	<0.001
Sad mood	13.8	0.33 (−0.31, 0.98)	0.311	4.2	0.10 (−0.12, 0.32)	0.375	26.7	0.64 (0.37, 0.92)	<0.001
Sexuality problems	−1.4	−0.05 (−0.96, 0.85)	0.91	9.8	0.34 (0.04, 0.64)	0.026	25.7	0.89 (0.52, 1.27)	<0.001
Cough	57.8	0.93 (0.33, 1.52)	0.002	11.2	0.18 (−0.02, 0.38)	0.081	47.8	0.77 (0.52, 1.03)	<0.001
Night sweats	−5.0	−0.12 (−0.82, 0.58)	0.728	15.8	0.38 (0.14, 0.62)	0.002	33.8	0.81 (0.51, 1.11)	<0.001
Itching	−8.6	−0.18 (−0.86, 0.5)	0.606	21.1	0.44 (0.21, 0.67)	<0.001	41.1	0.86 (0.57, 1.15)	<0.001
Bone pain	29.3	0.54 (−0.13, 1.21)	0.114	16.8	0.31 (0.08, 0.54)	0.007	53.3	0.98 (0.69, 1.26)	<0.001
Fever	38.7	0.12 (−0.15, 0.39)	0.376	0.0	0.00 (−0.09, 0.09)	0.939	29.0	0.09 (−0.03, 0.2)	0.142
Weight loss	176.6	2.19 (1.69, 2.68)	<0.001	−53.2	−0.66 (−0.83, −0.49)	<0.001	−52.4	−0.65 (−0.86, −0.44)	<0.001
Quality of life	25.9	0.75 (0.16, 1.33)	0.013	6.6	0.19 (0.00, 0.39)	0.055	17.9	0.52 (0.27, 0.77)	<0.001
Total Symptom Score	25.1	5.37 (1.47, 9.27)	0.007	6.4	1.36 (0.04, 2.67)	0.044	26.8	5.73 (4.07, 7.39)	<0.001

^1^ Compared with Normal weight.

## Supplementary Materials

The following are available online at https://www.mdpi.com/2072-6694/12/8/2202/s1, Figure S1: The MPN-SAF questionnaire. Table S1: BMI by MPN Subtype. Table S2: TSS by MPN Subtype and BMI.
