# Supplementary material for: Body Mass Index and Total Symptom Burden in Myeloproliferative Neoplasms Discovery of a U-shaped Association
Source: Cancers (Basel). 2020 Aug 6;12(8):2202. doi: 10.3390/cancers12082202 (PMC7465643; doi:10.3390/cancers12082202)
Supplement: Supplementary file 1 [file cancers-12-02202-s001.pdf]

Supplementary Materials: Body Mass Index and Total Symptom Burden in Myeloproliferative Neoplasms Discovery of a U-shaped Association

Sarah Friis Christensen, Robyn Marie Scherber, Nana Brochmann, Martin Goros, Jonathan Gelfond, Christen Lykkegaard Andersen, Esben Meulengracht Flachs, and Ruben Mesa

Myeloproliferative Neoplasm Symptom Assessment Form
(MPN-SAF) ©

**Circle the one number that describes how, during the past Week how much difficulty you have had with each of the following symptoms**	
Filling up quickly when you eat (Early satiety)	(Absent) 0 1 2 3 4 5 6 7 8 9 10 (Worst Imaginable)
Abdominal pain	(Absent) 0 1 2 3 4 5 6 7 8 9 10 (Worst Imaginable)
Abdominal discomfort	(Absent) 0 1 2 3 4 5 6 7 8 9 10 (Worst Imaginable)
Inactivity	(Absent) 0 1 2 3 4 5 6 7 8 9 10 (Worst Imaginable)
Problems with headaches	(Absent) 0 1 2 3 4 5 6 7 8 9 10 (Worst Imaginable)
Problems with concentration - Compared to prior to my MPD	(Absent) 0 1 2 3 4 5 6 7 8 9 10 (Worst Imaginable)
Dizziness/ Vertigo/ Lightheadedness	(Absent) 0 1 2 3 4 5 6 7 8 9 10 (Worst Imaginable)
Numbness/ Tingling (in my hands and feet)	(Absent) 0 1 2 3 4 5 6 7 8 9 10 (Worst Imaginable)
Difficulty sleeping	(Absent) 0 1 2 3 4 5 6 7 8 9 10 (Worst Imaginable)
Depression or sad mood	(Absent) 0 1 2 3 4 5 6 7 8 9 10 (Worst Imaginable)
Problems with sexual desire or Function	(Absent) 0 1 2 3 4 5 6 7 8 9 10 (Worst Imaginable)
Cough	(Absent) 0 1 2 3 4 5 6 7 8 9 10 (Worst Imaginable)
Night sweats	(Absent) 0 1 2 3 4 5 6 7 8 9 10 (Worst Imaginable)
Itching (pruritus)	(Absent) 0 1 2 3 4 5 6 7 8 9 10 (Worst Imaginable)
Bone pain (diffuse not joint pain or arthritis)	(Absent) 0 1 2 3 4 5 6 7 8 9 10 (Worst Imaginable)
Fever (>100 F)	(Absent) 0 1 2 3 4 5 6 7 8 9 10 (Daily)
Unintentional weight loss last 6 months	(Absent) 0 1 2 3 4 5 6 7 8 9 10 (Worst Imaginable)
What is your overall quality of life?	(As good as it can be) 0 1 2 3 4 5 6 7 8 9 10 (As bad as it can be)

© Mayo Clinic

Figure S1. The MPN-SAF questionnaire.

Table S1. BMI by MPN Subtype.

BMI category	**ET** ***n* = 1047)**	**PV** ***n* = 1301**	**MF** ***n* = 330**	**MPN-U** ***n* = 433**	**Total** ***n* = 3111**
BMI (Mean, sd)*	25.6 (5.2)	25.9 (5.1)	25.8 (5.2)	25.5 (5)	25.7 (5.2)
Underweight (N, %)	23 (2.2)	31 (2.4)	5 (1.5)	13 (3.0)	72 (2.3)
Normal Weight (N, %)	547 (52.2)	631 (48.5)	164 (49.7)	224 (51.7)	1,566 (50.3)
Overweight, (N, %)	316 (30.2)	423 (32.5)	111 (33.6)	132 (30.5)	982 (31.6)
Obese, (N, %)	161 (15.4)	216 (16.6)	50 (15.2)	64 (14.8)	491 (15.8)

* The BMI means did not differ significantly between the different MPN subtypes.

Table S2. TSS by MPN Subtype and BMI (mean, sd).

BMI category	**ET** ***n* = 1047**	**PV** **n = 1301**	**MF** ***n* = 330**	**MPN-U** ***n* = 433**	**Total** ***n* = 3111**
All BMI categories*	21.9 (16.6)	23.3 (17.2)	25.2 (16.4)	22.4 (15.9)	22.92 (16.8)
Underweight	25.5 (18.3)	26.7 (17.0)	41.0 (12.2)	23.1 (16.6)	26.7 (17.3)
Normal Weight	19.9 (16.2)	21.6 (16.6)	23.4 (16.2)	22.9 (16.2)	21.4 (16.4)
Overweight	21.9 (16.7)	23.67 (17.1)	24.4 (17.2)	19.1 (14.2)	22.6 (16.7)
Obese	28.0 (16.2)	27.4 (18.4)	31.2 (13.7)	27.3 (17.0)	28.0 (17.1)

* There is a significant difference in mean TSS between MF and ET patients ($p < 0.05$).

© 2020 by the authors. Licensee MDPI, Basel, Switzerland. This article is an open access article distributed under the terms and conditions of the Creative Commons Attribution (CC BY) license (http://creativecommons.org/licenses/by/4.0/).
